# Primary pancreatic hydatid cyst: A rare case report and diagnostic challenges

**DOI:** 10.1002/ccr3.9162

**Published:** 2024-07-08

**Authors:** Shokouh Taghipour Zahir, Amirhossein Rafiee, Saeed Kargar

**Affiliations:** ^1^ Shahid Sadoughi University of Medical Sciences Yazd Iran

**Keywords:** *Echinococcus granulosus*, hydatid cyst, pancreatic cyst, Whipple's surgery

## Abstract

**Key Clinical Message:**

In cystic lesions of the pancreas, hydatid cyst should be considered in the differential diagnoses and its presence should be ruled out before any invasive interventions. Serological tests along with imaging studies related to hydatid cyst diagnostic indicators should be performed in people who live in *Echinococcus granulosus* endemic areas and suffer from cystic lesions of the gastrointestinal tract.

**Abstract:**

Primary pancreatic hydatid cysts, caused by the tapeworm *Echinococcus granulosus*, represent a rare occurrence often challenging to diagnose due to their similarity to other pancreatic conditions. This case report outlines a 67‐year‐old male presenting with jaundice and cholestasis but lacking typical symptoms associated with pancreatic hydatid cysts. Laboratory findings revealed elevated bilirubin levels, liver enzyme abnormalities, and tumor markers, prompting imaging studies that indicated a cystic mass near the pancreatic head. Misdiagnosed initially as a mucinous cystic neoplasm, the patient underwent Whipple surgery, unveiling a large cystic lesion upon examination.

## Graphical abstract

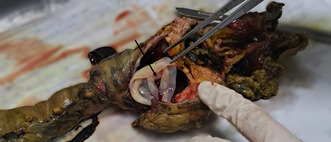

## 1 INTRODUCTION

The hydatid cyst is a zoonosis caused by the tapeworm *Echinococcus granulosus*.^1^ It is a major problem in developing countries such as Iran and is found in the middle east, India, Australia and Turkey more than anywhere else.^2^ Dogs are considered the definitive host and sheep and goats are intermediate hosts.^3^ Humans are the accidental hosts.^1^ Primary pancreatic hydatid cyst (PHC) is one of the rarest variants (0.14%–2%).^4^ It is usually diagnosed by accident as for the pancreas itself, the parasite affects the head the most (57%), the body (24%) and tail (17%) respectively.^5^, ^6^ Most of the hydatid cysts affecting the abdomen are asymptomatic.^7^ However the most common symptoms are LUQ or epigastric pain, nausea, vomiting and fever that come with mass effect symptoms based on the location.^7^, ^8^ PHC often causes obstructive jaundice and some of the other complications are cholangitis, rupture of the cyst, pancreatitis, abscess formation and fistula.^6^ The disease is often misdiagnosed if it is not clinically suspected at first.^6^ Few case reports of pancreatic hydatid cysts have been published up to now. Herein we present a rare case of pancreatic head and Ampulla of Vater hydatid cyst with review of literature.

## 2 CASE HISTORY AND EXAMINATION

A 67‐year‐old male patient came in our clinic with jaundice and cholestasis without a history of nausea, vomiting, fever, weight loss, or altered bowel habits. Laboratory findings revealed elevated bilirubin levels, liver enzyme abnormalities, and tumor markers (Table 1).

**TABLE 1:** Laboratory Findings (both CA 19–9 measurements were before the surgery).

Total Bilirubin	6.6 mg/dL	Direct Bilirubin	4.2 mg/dL
AST	100 IU/L	ALT	157 IU/L
ALP	969 U/L	Amylase	125 IU/L
Lipase	115 IU/L	CEA	4.94 ng/mL
CA 19–9	43.10 U/mL	CA 19–9	60.59 U/mL

## 3 METHODS (DIFFERENTIAL DIAGNOSIS, INVESTIGATIONS AND TREATMENT)

**FIGURE 1:**
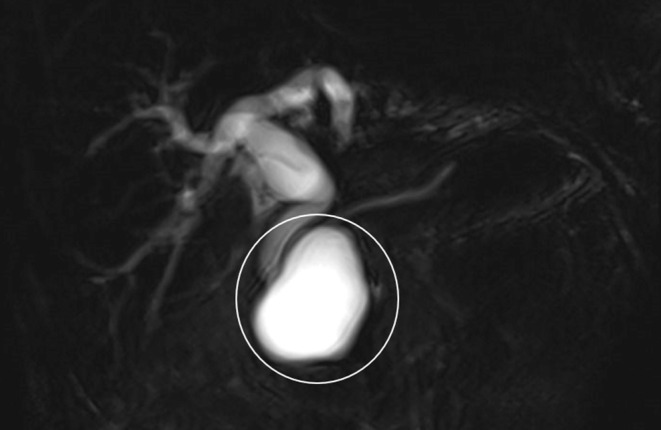
MRCP showed a 64 mm × 40 mm cystic structure adjacent to distal CBD.

**FIGURE 2:**
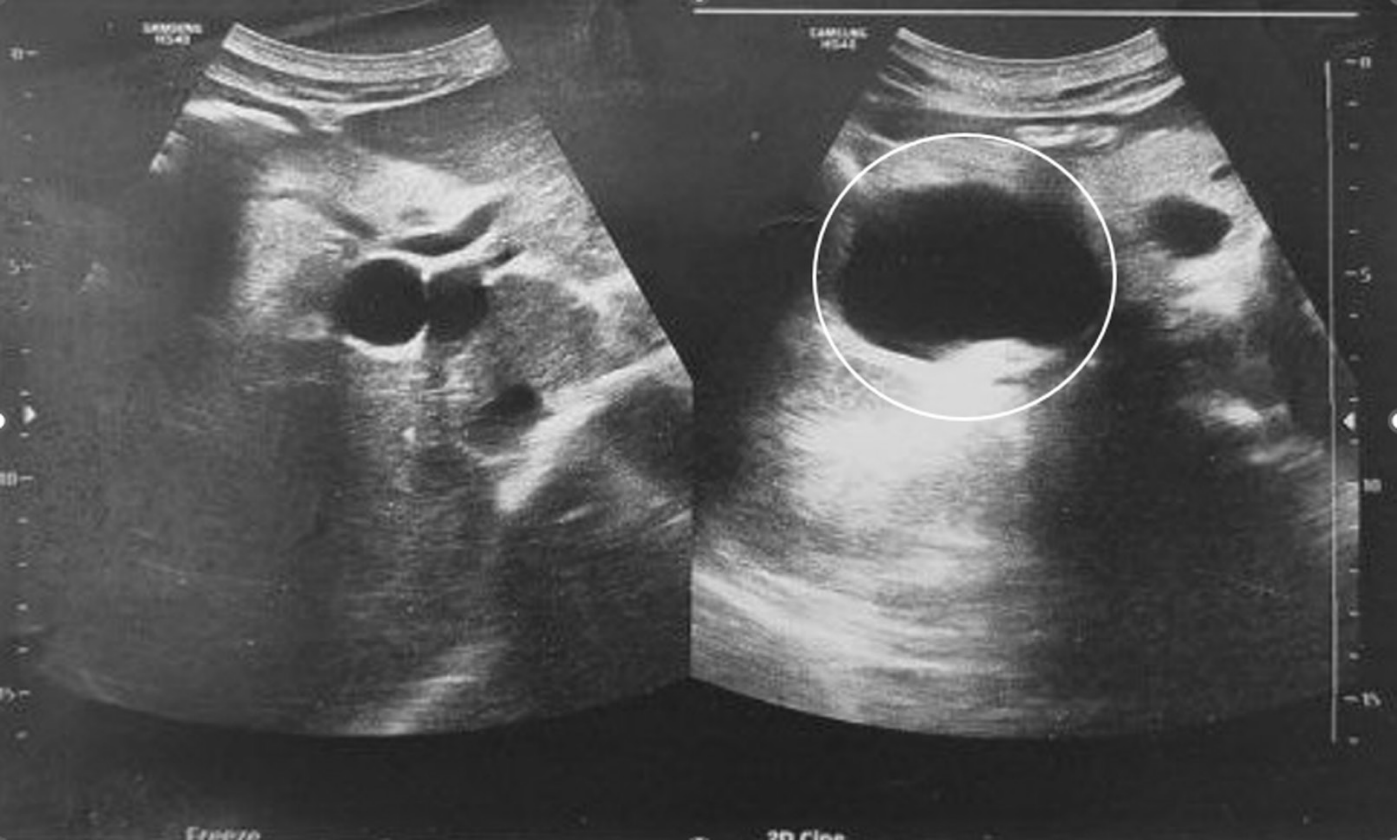
Endoscopic Ultrasonography suggested a large cystic thick wall lesion at the pancreatic head area. (white circle).

**FIGURE 3:**
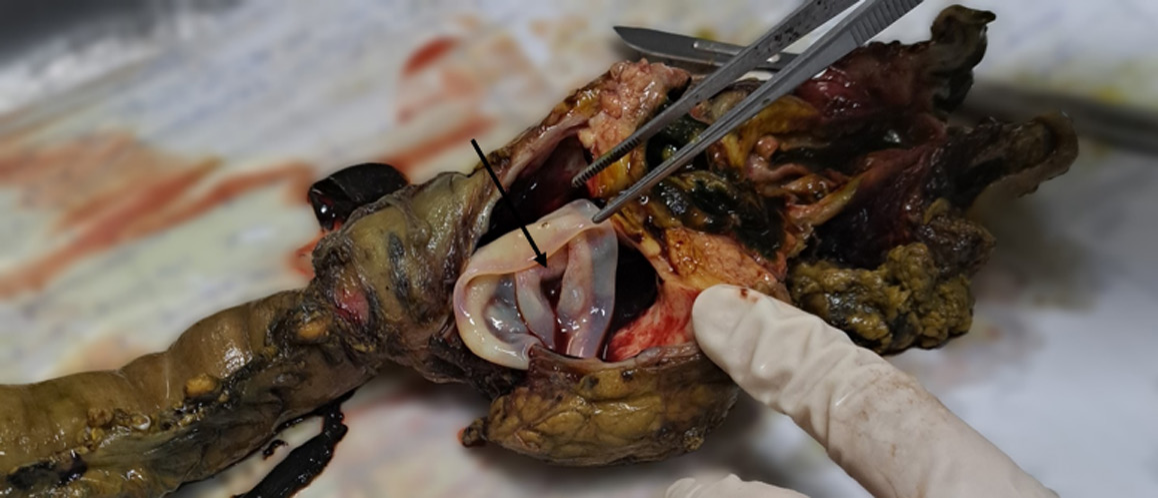
Macroscopic examination of the resected specimen reveals cystic lesion in the head of the pancreas. (Arrow: Cyst wall).

**FIGURE 4:**
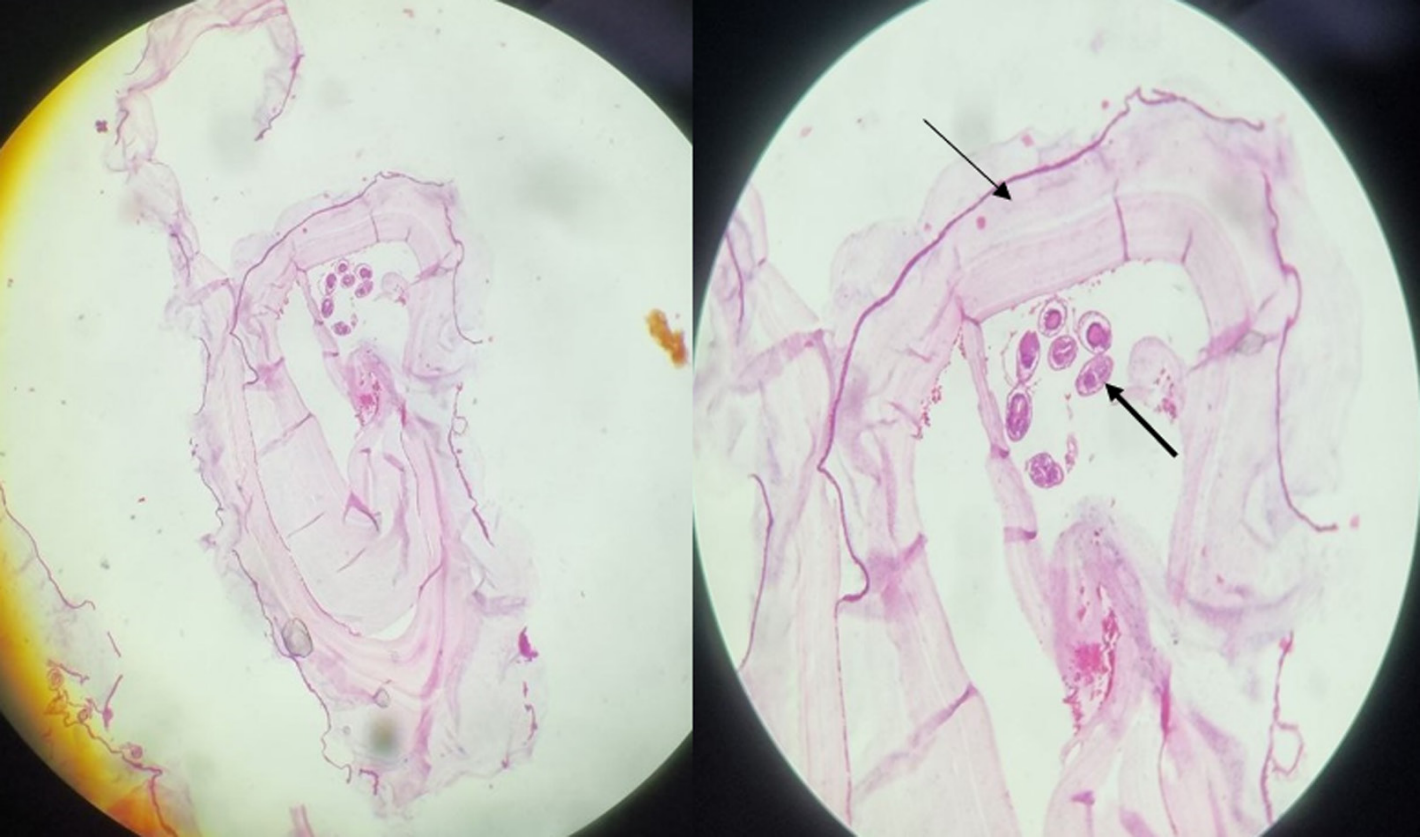
Microscopic examination of the cyst wall shows inner germinal; layer, outer lamina lucida layer. (x20 magnification) (thin arrow: Cyst wall / thick arrow: Scolices).

Abdominopelvic Ultrasonography in another medical center had shown a 42 mm × 66 mm cystic mass in the posterior region of pancreatic head with dilated intra and extra hepatic biliary ducts and a dilated common bile duct (CBD) (22 mm) with a 9 mm × 12 mm hypoechoic region on the distal part CBD. No gallbladder or liver pathology had been reported. Magnetic resonance (MR) cholangiopancreatography showed a 64 mm × 40 mm cystic structure adjacent to distal CBD containing some thick content that may be due to choledochal diverticula with a mildly dilated gallbladder containing sludge and dilated intra and extra hepatic biliary ducts and a CBD diameter of 21 mm (Figure 1). Endoscopic Ultrasonography (EUS) suggested a large cystic thick wall lesion at the pancreatic head area which was measured 60 mm × 44 mm in diameter without connection to main PD or solid component with compressive effect on CBD and dilated CBD. EUS feature was suspected for MCN (mucinous cystic neoplasm of the pancreas) (Figure 2). EUS guided aspiration fluid of the cyst content didn't reveal specific pathology. Based on these findings the patient was a candidate for Whipple surgery. Received specimen consisted of pancreas (8 cm × 8 cm), part of stomach, gallbladder, part of small intestine and resected peripancreatic abdominal lymph nodes. On section there was a 6 cm × 6 cm cystic lesion in the head of pancreas (Figure 3). Microscopic examination revealed cyst wall composed of inner germinal layer with daughter cysts and scolices, outer lumina lucida acellular layer and beyond that the host's fibrotic reaction (Figure 4). Resected part of small intestine (duodenum) and part of stomach were unremarkable. Finally, the diagnosis of pancreatic hydatid cyst was confirmed. After surgery, our patient was put on medical treatment by albendazole (400 mg twice per day for 1 month).

## 4 CONCLUSION AND RESULTS (OUTCOME AND FOLLOW‐UP)

In conclusion, based on this case report, we recommend that this disease be considered in the differential diagnosis of gastrointestinal cysts, especially those involving the pancreas, liver, and the ampulla of Vater, particularly in endemic areas for Echinococcus, so that first, unnecessary surgical intervention can be avoided, and then if surgery is necessary, appropriate prophylaxis could be administered to prevent complications related to the cyst and its rupture, such as fistula formation, etc. Although our patient was not diagnosed before the surgery, fortunately, the area containing the cyst was removed intact and without tearing, and the subsequent complications that could have caused a fistula were prevented. After appropriate treatment, now imaging studies and laboratory studies are within normal limits.

## 5 DISCUSSION

Pancreatic cysts can range from asymptomatic types discovered accidentally to symptomatic cases.^1^, ^2^ Most pancreatic cysts are pseudocysts, followed by mucinous and serous cysts.^3^, ^4^ Hydatid cysts are considered the rarest cystic diseases of the pancreas and may be diagnosed during ultrasound studies. If they are not diagnosed by imaging studies, rupturing and fistula formation during invasive approaches is possible.^7^, ^8^, ^9^ A primary pancreatic hydatid cyst (PHC) represents one of the least common forms (0.14%–2% occurrence).^4^ Typically, it's incidentally detected during diagnostic procedures and is often not accompanied by the secondary form of the disease.^3^, ^5^ Regarding the pancreas, the parasite predominantly impacts the head (57%), followed by the body (24%) and the tail (17%).^6^ Primary pancreatic hydatid cysts frequently lead to obstructive jaundice.^6^ Aspiration cytology of cyst fluid reveals the scolices in the smear to avoid unnecessary and invasive surgeries.^10^, ^11^ Before surgery, our patient had EUS‐guided cyst fluid aspiration, but the cytology examination did not reveal hydatid scolices. Due to the pressure and space‐occupying effect of the hydatid cyst on the pancreatic duct, the elevation of pancreatic enzymes and tumor markers was reported. Unlike the case reported by Hasnaouia A and his colleagues, that there was a hydatid cyst in the tail of the pancreas and they were able to diagnose it through imaging because the patient lived in an endemic area before surgery.^12^ Therefore, the patient was treated before the surgery. In this case, hydatid cyst was not considered as a differential diagnosis among the clinical and ultrasound studies, and the lesion was considered an epithelial pancreatic cyst such as a mucinous cyst, so invasive Whipple surgery was performed. Uncommon complications associated with hydatid cysts located in the pancreatic head include conditions like cholangitis, acute and chronic pancreatitis, duodenal stenosis or fistula, and the formation of pancreatic abscesses. Cysts found in the body and tail of the pancreas typically do not show symptoms and might only be identified due to the presence of an abdominal mass.^4^ The disease is often misdiagnosed if it's not clinically suspected in the first investigation step.^6^ Diagnosis of hydatid cysts can be done by imaging such as ultrasonography, CT scan, MRI, or by serologic tests such as ELIZA for the parasite's antigens.^2^, ^9^ Findings in ultrasonography are the most sensitive for cyst membrane, septa, and hydatid sand but CT scans and MRI are less viable in this regard.^7^ Despite these methods, postoperative histopathological examination remains the gold standard for diagnosis.^6^ Degeneration of the cyst wall, concomitant by the development of daughter cysts appearing as nested cysts separated by the hydatid matrix (comprising membranes, hydatid fluid, and hydatid sand) of varied echogenicity, can occasionally result in imaging findings resembling those seen in mucinous cystadenomas. While most mucinous cystadenomas (MCNs) are multi‐compartmental, a single‐compartment appearance is also observable. In this report, the PHC was misdiagnosed as mucinous cystadenoma similar to what Mitrovic and colleagues had reported in 2020.^4^ As cystic echinococcosis progresses, the cyst changes its structure. Surgery is commonly regarded as an effective treatment method.^9^, ^10^ Additionally, administering anti‐helminthic medication such as Albendazole at a dosage of 10–15 mg/kg/day for 2–4 weeks before surgery, and continuing it for 4 weeks post‐surgery, has been suggested to decrease the risk of recurrence.^4^ The surgical approach recommended varies based on the cyst's location and its connection to the main pancreatic duct. Surgeons may consider procedures such as cyst fenestration, partial pericystectomy, or in instances where there's communication with the main pancreatic duct, more extensive surgeries like Whipple's pancreaticoduodenectomy or distal pancreatectomy.^11^, ^12^ The choice of procedure depends on factors like cyst location and the nature of its connection to the pancreatic duct.^10^

## AUTHOR CONTRIBUTIONS

**Shokouh Taghipour Zahir:** Conceptualization; formal analysis; methodology; project administration; supervision; validation; writing – review and editing. **Amirhossein Rafiee:** Data curation; investigation; writing – original draft. **Saeed Kargar:** Data curation; methodology; project administration; resources; validation.

## CONFLICT OF INTEREST STATEMENT

The authors have no conflict of interest to declare.

## CONSENT

The authors state that written and informed consent considering the use of the patient's clinical information and images was obtained prior to the admission of this manuscript.

## ETHICS STATEMENT

Ethical approval code: IR.SSU.MEDICINE.REC.1402.345.

## Data Availability

All the documents and evidence of the patient are available.
